# Spread of Antimicrobial Resistance by *Salmonella enterica* Serovar Choleraesuis between Close Domestic and Wild Environments

**DOI:** 10.3390/antibiotics9110750

**Published:** 2020-10-29

**Authors:** María Gil Molino, Alfredo García, Sofía Gabriela Zurita, Francisco Eduardo Martín-Cano, Waldo García-Jiménez, David Risco, Joaquín Rey, Pedro Fernández-Llario, Alberto Quesada

**Affiliations:** 1 Unidad de Patología Infecciosa, Facultad de Veterinaria, Universidad de Extremadura, 10003 Cáceres, Spain; sofigz32@gmail.com (S.G.Z.); jmrey@unex.es (J.R.); 2 Área de Producción Animal, CICYTEX-La Orden, 06187 Badajoz, Spain; fredgarsa@gmail.com; 3 Unidad de Reproducción, Facultad de Veterinaria, Universidad de Extremadura, 10003 Cáceres, Spain; femartincano@unex.es; 4 Innovación en Gestión y Conservación de Ingulados S.L. (INGULADOS), 10004 Cáceres, Spain; waldo@ingulados.com (W.G.-J.); david@ingulados.com (D.R.); pedro@ingulados.com (P.F.-L.); 5 Neobeitar S.L., 10004 Cáceres, Spain; 6 Departamento de Bioquímica, Biología Molecular y Genética, Facultad de Veterinaria, Universidad de Extremadura, 10003 Cáceres, Spain; aquesada@unex.es; 7 INBIO G+C, Universidad de Extremadura, 10003 Cáceres, Spain

**Keywords:** *Salmonella Choleraesuis*, Iberian pig, wild boar, antibiotic resistance, phylogenetic relationship, plasmid replicon typing, colistin

## Abstract

The *Salmonella* *enterica* serovar Choleraesuis affects domestic pig and wild boar (WB), causing clinical salmonellosis. Iberian swine production is based on a free-range production system where WB and Iberian pig (IP) share ecosystems. This study focuses on the negative impact on the pork industry of infections due to this serotype, its role in the spread of antibiotic resistance, and its zoonotic potential. Antibiotic resistance (AR) and genetic relationships were analyzed among 20 strains of *S. Choleraesuis* isolated from diseased WB and IP sampled in the southwest region of the Iberian Peninsula. AR was studied using the Kirby–Bauer method with the exception of colistin resistance, which was measured using the broth microdilution reference method. Resistance and Class 1 integrase genes were measured using PCR, and the genetic relationship between isolates and plasmid content by pulsed field gel electrophoresis. The results show a higher incidence of AR in isolates from IP. Phylogenetic analysis revealed seven profiles with two groups containing isolates from IP and WB, which indicates circulation of the same clone between species. Most pulsotypes presented with one plasmid of the same size, indicating vertical transmission. AR determinants *bla_TEM_* and *tetA* were routinely found in IP and WB, respectively. One isolate from IP expressed colistin resistance and presented the *mcr-1* gene carried by a plasmid. This study suggests that *S. Choleraesuis* circulates between WB and IP living in proximity, and also that the mobilization of AR genes by plasmids is low. Furthermore, the detection of plasmid-mediated colistin resistance in bacteria from IP is alarming and should be monitored.

## 1. Introduction

Salmonellosis in swine results in tremendous economic losses in the pork industry [1]. *Salmonella enterica* subsp. *enterica* serovar Choleraesuis (*S.* Choleraesuis) causes clinical salmonellosis in pigs and wild boar (WB) [2], and the identification of epidemiologic groups strongly suggests an exchange of this serovar between WB and domestic pigs [3]. Nowadays, *S. Choleraesuis* is still very common in North America and Asia and, although it is not considered a dominant serovar in pigs from Europe [4,5], different outbreaks have occasionally been reported in recent years [6,7] including in WB [2,3,8,9,10,11].

The Iberian pig (IP) is an autochthonous breed that originated in the Iberian Peninsula, for which the production system is mainly associated with extensive management deeply linked to the Mediterranean ecosystem and traditional agroforestry in the southwest of the Iberian Peninsula [12]. This means that WB and IP share the same habitats, leading to subsequent interactions among them [13,14]. WB, as an omnivorous species, is prone to multiple pathogen exposure. They have been shown to carry resistant bacteria [15] and could be a gateway for spread of this resistance from domestic animals or humans to wildlife [16]. Besides, several studies have shown WB as a possible asymptomatic persistent reservoir of *S.* Choleraesuis [17,18].

Although *S.* Choleraesuis is swine-specific and rarely infects other hosts, it is the second most predominant serovar among human isolates in Taiwan and exhibits the highest degree of invasiveness [19,20], which may result in severe disease and death [21]. Most *S.* Choleraesuis isolates from humans and swine exhibit closely related DNA fingerprints, indicating that human infections were acquired from pigs [22], reinforcing the importance of controlling this serotype in Suidae.

Most *S.* Choleraesuis strains that have caused infections in humans, mainly in Asian countries, are multidrug resistant (MDR) [19,23], which has been associated with classical mobile genetic elements (i.e., transposons and plasmids) and integrative elements that can spread antimicrobial resistance genes within the bacterial host genome through gene cassettes by site-specific recombination [24,25]. In addition, plasmids can carry other gene functions such as those involved in virulence by pSCV50 in *S. Choleraesuis* [26]. This 50 kb plasmid does not carry antimicrobial resistance genes, although it can recombine with larger sized plasmids detected in *S. Choleraesuis* where *sul1*, *bla*_TEM_, and extended-spectrum beta-lactamase genes are located [27,28,29].

In contrast to the limited administration of colistin (polymyxin E) to humans as a last resort antibiotic, it has historically been used for prophylaxis in animal production [30]. Consequently, a dramatic increase of colistin resistance has arisen in naturally sensitive Gram-negative bacteria, with the spread of plasmid carrying *mcr-1* among other resistance determinants [31]. Among different reservoirs, livestock is considered the main source of *mcr* genes worldwide [32], and a global concern exists due to their high mobilization potential by plasmids carrying other resistance determinants [33]. *S*. *enterica*, one of the most clinically relevant enterobacteria, carries colistin resistance genes in many serovars via different plasmids, including IncHI2 mega-plasmids larger than 200 kb with multiple resistance determinants [34]. In *S.* Choleraesuis, this has been described very recently in one MDR isolate from a human blood infection in Brazil, linked to a 40 kb IncX4 plasmid [35].

The aim of the present investigation was to study the genetic relationship between strains of *S. Choleraesuis* from IP and WB raised in the southwest of the Iberian Peninsula and to address the mechanism of spread of its antimicrobial resistance determinants, including through screening for low-susceptible isolates to colistin in this bacterial pathogen.

## 2. Results

### 2.1. Clustering of S. Choleraesuis Isolates by PFGE-XbaI

**Figure 1:**
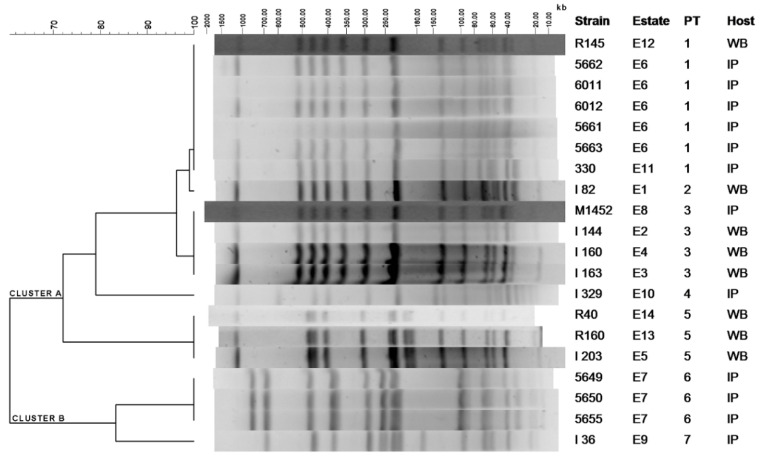
Dendrogram based on PFGE macrorestriction pattern of *S. Choleraesuis* isolates. Dendrogram showing 7 different profiles (PT) further divided into two clusters A and B. Dice coefficients had a 1.5% band position tolerance. The scales at the top indicate the similarity indices (in percentages) and molecular sizes (in kilobases).

Pulsed-field gel electrophoresis (PFGE) (*XbaI*) macrorestriction displayed seven different profiles or pulsotypes (PT) grouped into two main clusters: A, with a degree of similarity higher than 75% and B, with more than 80% similarity (Figure 1). Whilst cluster B contains only 4 isolates from 2 estates, all of them from IP, cluster A groups 5 PT that contain 15 isolates from 12 different estates. Within this cluster PT1, PT2 and PT3 showed a degree of similarity higher than 95%. There is remarkable persistency over time for PT1, PT3, and PT5, which were isolated during 5, 3, and 4 year periods, respectively, from the animal populations. Among them, PT1 and PT3 were detected in both IP and WB, indicating bacterial circulation between both suids. The distance between the estates with the same PT was not significantly different than the average distance between all the estates included in the study.

### 2.2. Resistance Determinants against Clinically Relevant Antimicrobials in the S. Choleraesuis Isolates

Resistance against at least one of the 14 tested antibiotics was found in almost all tested strains (19/20; 95%); moreover, 65% (13/20) of the *S. Choleraesuis* isolates were multidrug resistant (MDR) with resistance to 4 or more antibiotics (Table 1). Antimicrobial resistance phenotypes were highly variable, with 14 different patterns existing among the 20 *S. Choleraesuis* isolates (Table 1), even within the same PT, especially if they came from different estates, as observed in PT1 and PT5 (Figure 1). Only three patterns appeared more than once: AMP–TRS–SUL–CHL (3 isolates from PT6), AMP–STR–TRS–SUL (2 isolates from PT1), and NEO (2 isolates from PT3), and none of them were shared between IP and WB. Indeed, the average number of antimicrobials to which isolates presented resistance depended on the host, with 4.9 resistances (or MDR), on average, per isolate in IP and 2.8 in WB. The host effect on MDR of isolates also affects the particular antibiotics found in every spectrum. Among isolates from IP, the most common resistance observed is against ampicillin, followed by sulfonamide, while in those from WB, the lowest susceptibilities were found against aminoglycosides (streptomycin and neomycin) followed by tetracycline and sulfonamide (Table 2). Resistance against colistin, a last resort antibiotic in human health, is found in only one isolate of PT1 from IP. Regardless of their origin, all isolates were susceptible to quinolones or the broad-spectrum cephalosporin cefotaxime.

Antimicrobial resistance determinants were found in all the strains from IP and most (seven out of eight) from WB. The antimicrobial resistance genes detected were highly variable among isolates, with a total of 10 different genotypes, 50% of them with four or more resistance genes (Table 1). Similarly to antimicrobial-resistant phenotypes, genotypes were variable among isolates, even from the same PT, with *bla*_TEM_, *bla*_TEM_–*aadA1–sul3*, and *strA–strB–tetA* found most frequently (Table 1). Considering each resistance gene, the β-lactamase-encoding *bla*_TEM_ was most common with nine strains from IP and two from WB, covering all PT except PT5. However, from WB the most prevalent was *tetA*, found in one isolate from PT1 and three from PT5 in addition to only one isolate from PT3 in IP. The *int1* gene, encoding the class 1 integrase that is frequently linked to antimicrobial resistance gene cassettes, was detected in five isolates, all from IP, although two of them share PT with WB isolates (PT1 and PT3, Figure 1). However, only two isolates presented *int1*-linked gene cassettes of 1000 or 1200 bp length which also coded for *aadA2* or *bla*_PSE1_ genes, respectively (Table 1).

Interestingly, the *mcr-1* (plasmid-mediated colistin resistance) gene was detected in one colistin-resistant isolate from IP belonging to PT1, the most common PT among *S. Choleraesuis* isolates (Table 1). In general, isolates carrying resistance determinants presented low susceptibility to the corresponding antimicrobial(s), although aadA1 and *strA* genes may be expressed weakly or not at all.

### 2.3. Plasmid Content of S. Choleraesuis Isolates

**Figure 2:**
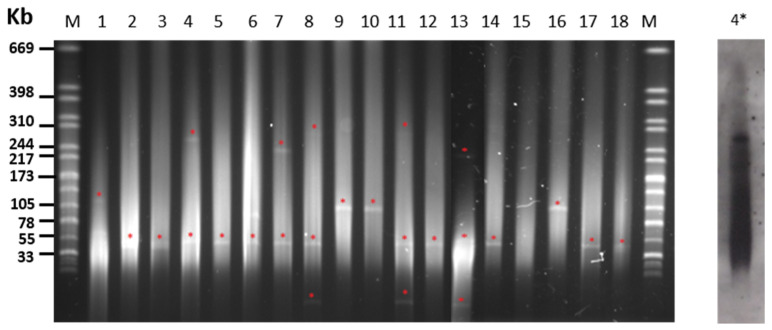
Plasmid analysis of *S.* Choleraesuis isolates from Iberian pigs and wild boar in Spain. PFGE-S1 analysis of isolates. Red asterisks indicate plasmids for which an approximate size has been estimated by comparison with *S. braenderup* molecular weight standards. Analyzed isolates are **M**, *S. braenderup* digested by XbaI; **1**, R145; **2**, 6011; **3**, 5661; **4**, I330; **5**, I160; **6**, I163; **7**, I329; **8**, I203; **9**, 5655; **10**, 5650; **11**, R40; **12**, M1452; **13**, R160 **14**, I82; **15**, 36; **16**, 5649; **17**, I144; **18**, 5662. 4* Hybridization to a DIG-labeled *mcr-1* probe.

S1 nuclease treatment and PFGE typing of plasmid content revealed that 19 out the 20 strains carried at least one extrachromosomal molecule of DNA, with five isolates carrying multiple plasmids (Table 1 and Figure 2). The plasmid most frequently found was 50 kb in size, shared by 75% of isolates, including those fully sensitive to antibiotics and lacking resistance genes. Plasmids between 100 and 300 kb were also detected in strains mostly expressing MDR. Due to its clinical relevance, plasmid location was performed for the colistin-resistance *mcr-1* gene identified in this study for the first time in *S. Choleraesuis* isolated from swine (Figure 1). Thus, a plasmid slightly over 240 kb in size was detected that was carrying *mcr-1* from an IP necropsied in 2020, as revealed by specific hybridization with a DIG-labeled probe from a previously characterized sequence [36]. With exceptions, as for the mentioned plasmid carrying *mcr-1* in a PT1 strain, the number and size of plasmids was found to be stable in isolates within every PT.

## 3. Discussion

**Figure 3:**
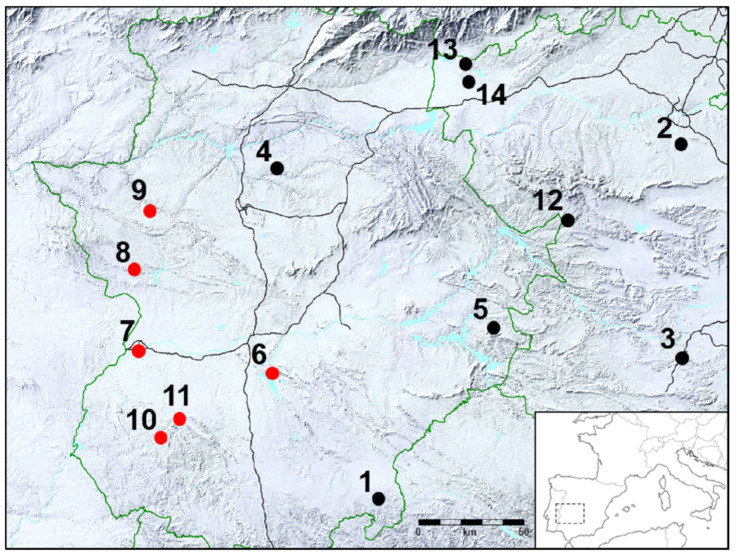
Location of the estates. Geographical map of the southwest Iberian Peninsula displaying the central location of the different estates from where *S.* Choleraesuis suid hosts were sampled. Black dots represent estates where WB were sampled and red dots IP farms. Black lines represent highways and green lines administrative division limits (inset: location of the Iberian Peninsula in southwestern Europe).

In this study, isolates of *S. Choleraesuis* from IP and WB have been analyzed in order to trace the spread potential of antimicrobial resistance determinants carried by this serotype in the “dehesa”, a traditional agrosystem consisting of grassland with Holm’s oaks found in the Iberian Peninsula. The XbaI-PFGE profile of *S.* Choleraesuis isolates revealed different PT, but most of the strains (16/20) belonged to the same cluster with a degree of similarity above 75% (Cluster A), among which PT1, PT3, and PT5 might represent clones with high potential spread both in space and time, in agreement with previous studies in WB [2,3,17] and domestic pigs [5,6]. With regard to phylogeographic analysis, a recent study demonstrated cross-border transmission of *S. Choleraesuis* from pigs between countries that was concordant with the trading network [18]. In our study, genetic relationships were detected not only among bacteria from the same species, but also with the wild ancestor of pigs, the WB, which share the “dehesa” environment with IP [14]. In our study, the geographical link between animals is maximal for WB from estates E4 and E1, the closest to IP farms E6 and E11 (Figure 3) from which *S. Choleraesuis* isolates share PT1, PT2, or PT3 in closely related backgrounds (>95%, Figure 1). On the other hand, it should be noted that there are large distances between these estates; approximately 70 km between E6 and E1 and all of them (E1, E4, E6, and E11) in a radius of 90 km (Figure 3). Apart from the distance, the estates are also separated by several highways (E11 and E4) and a large river (E4). Moreover, one WB isolate from a faraway estate, E12, also shares PT1. When considered together, all these facts suggest that proximity itself is not the main reason for the bacterial relationship and that other factors may be responsible, i.e., human carriers or animal trading, although evidence is lacking. Together with studies showing the spread of *S.* Choleraesuis between WB and domestic pigs [3,18], including asymptomatic WB in Europe [17,37,38], our results show a wildlife reservoir that may spill over to farmed pigs or vice versa.

MDR was detected in 83.3% (10/12) of isolates from IP in this study, higher than the 37.5% (3/8) observed in WB. Similar prevalences of antimicrobial resistance have been reported in *S. Choleraesuis* from domestic pigs in Asia [26,39] but these are higher than previous reports in Europe [5,6]. The data from WB are similar to those previously described by our group [2]. Likewise, the antibiotic groups with higher resistances differ between *S. Choleraesuis* from the analyzed Suidae, showing resistance to ampicillin and sulfonamide for bacteria from IP, and sulfonamide, tetracycline, and streptomycin from WB, although streptomycin resistance had the same ratio in bacteria from both hosts, similarly to previous reports [2,3,6,40,41]. The lack of resistance found against quinolones and cephalosporins is in accordance with most of the studies from Europe [18,42], although outbreaks or sporadic cases of infections caused by *Salmonella* spp. with resistance to these antibiotics are being increasingly reported [39,43].

Isolates of *S. Choleraesuis* from the two hosts screened in this study, IP and WB, presented quantitative differences in antibiotic resistance found against ampicillin and trimethoprim/sulfamethoxazole, which are higher in the autochthonous pig breed. In contrast, resistance to chloramphenicol, gentamicin or colistin was only detected in IP. This could be due to the fact that many of these antibiotics have been extensively used as growth promoters (beta-lactams) or as prophylactic agents for common diseases such as colibacillosis (colistin) or coccidiosis (sulfonamides) in pig farms for a long time [44], which has been associated with increases in resistant bacteria [45]. Although the IP production system is linked to the dehesa in the last period of fattening, the first stages of breeding mostly take place on farms with semi intensive management systems. It was in these stages where antibiotic abuse has taken place in the past. Considering that frequent use has a stronger association with resistance than sporadic use [46,47], it could explain the lower number of resistances found in WB, as the treatments applied to them, when applied, are scarce and limited to certain short periods of time, which is different to the IP, especially in the early stages of breeding. However, even on estates that did not apply any antibiotic treatment, antibiotic resistances were found in *S. Choleraesuis* from WB. This could be due to the omnivorous behavior of WB, which means they visit communal refuse sites as well as the proximity of farmed animals like IP in free range production systems, where horizontal transmission of bacteria might occur [48,49].

Resistance genes have been previously detected in *S.* Choleraesuis from pigs and humans [18,28,41], but information is scarce in WB [2]. In this study, we described Class 1 integrons in 42% of the *S. Choleraesuis* isolates from IP and none in WB. Around 41% of these integrons carried a resistance gene cassette. These genetic elements play an important role in the development of antibiotic resistance and have a worldwide distribution in Gram-negative bacteria, colonizing both humans and animals [50]. In *S.* Choleraesuis from pigs, finding these elements in a large number of isolates is very common [39,51]. Interestingly, our study shows that the *sul3* gene occurs in 3 out of 5 Salmonella isolates carrying class 1 integrons, although the presence of this integron is more frequently related to the spread of the *sul1* gene [52,53].

Our study reveals that, with exceptions, *S.* Choleraesuis strains from IP or WB carry plasmids which are around 50 kb in size (Figure 1), that isolates lacking antimicrobial resistance did not present additional plasmids, and that bacteria expressing multiple antimicrobial resistance share mega-plasmids, alone or in addition to the 50 kb bands (Table 1). The fact that only closely related isolates share plasmid bands and/or antimicrobial resistance patterns might suggest that clonal spread prevails over horizontal transfer as the common mechanism for dispersion of antimicrobial resistance determinants in the analyzed environment. This study also shows the presence of the colistin-resistant gene *mcr-1* in one of the isolates studied from IP. In this strain, *mcr-1* is carried by a high-molecular weight plasmid (>240 kb), possibly conferring MDR and most probably belonging to the IncHI2-type replicon (different to the recent finding in a human isolate) [35], which could represent a risk for accumulation and/or spread antimicrobial resistance determinants through food chain environments, as for Iberian pigs, and their processed products and humans. Although more studies are needed to determine its prevalence, due to its clinical importance in human health, the presence of these colistin-resistant Salmonella isolates should be monitored in order to control their evolution.

## 4. Materials and Methods

### 4.1. Bacterial Strains and Animal Sources

The 20 strains of *S. Choleraesuis* isolated from diseased WB (*n* = 8) and IP (*n* = 12) were analyzed at the Clinical Veterinary Hospital (CVH) at the University of Extremadura. The animals were submitted to the CVH by veterinarians or by a hunting management company (Ingulados S.L.) from Cáceres, Spain, in order to determine the cause of death and control disease on their farms/game estates. After routine necropsy and microbiological analysis, those animals with *S.* Choleraesuis were included in the study. Each isolate from WB was derived from a different outbreak (clinical disease in several animals in a short period of time) and estate (E1–E11), whilst IP belonged to six different estates, among which several animals from the same outbreak were sampled in E6 and E7. All fourteen estates were located in the Central West region of the Iberian Peninsula (Figure 3). The IP estates were either breeding farms connected to a large enclosure of the dehesa ecosystem or just an enclosure where IP underwent a fattening process. The fences or walls that surround those enclosures are strong enough to keep the IP inside, but not enough to prevent the entry of WB and their subsequent interactions with IP. The WB came from game estates where they were occasionally fed and subjected to periodical health inspections, where they are captured, analyzed, and returned to their natural environment.

The clinical isolates came from different organs (liver, kidneys, lungs, and spleen) and were cultured on blood agar, MacConkey agar, and xylose–lysine–deoxycholate agar (XLD) under aerobic conditions for 24 h/37 °C. Colonies compatible with *Salmonella* were confirmed using conventional microbiological methodologies and identified as *Salmonella enterica* serovar Choleraesuis based on *fliC* gene PCR [54].

### 4.2. Pulsed-Field Gel Electrophoresis (PFGE) Analysis

Determination of the dendrogram of PFGE clusters among isolates of *S.* Choleraesuis was performed by macrorestriction with XbaI followed by PFGE (Chef-DR^®^III. Bio-Rad; Hercules, CA, USA) according to the PulseNet protocol with pulses oscillating from 2.16 to 63.8 s for 21.5 h [55], and *S. braenderup* was used as the molecular weight standard. The gel was stained with ethidium bromide, and DNA bands were visualized with an UV transilluminator. Images were prepared using Quantity One software (Bio-Rad; Hercules, CA, USA). The different PFGE profiles (PT) were analyzed by InfoQuest FP Software (Version 4.5).

Plasmid size analysis was performed by PFGE under the same conditions described above after incubation of plugs with S1 nuclease (Thermo Fisher, Waltham, MA, USA) according to manufacturer’s recommendations. For plasmid hybridization, PFGE was transferred to a nylon membrane and hybridized to a digoxigenin-labeled *mcr-1* probe that was PCR amplified from a previously described *E. coli* strain [36]. Digoxigenin labeling and detection were performed according to manufacturer’s instructions (Merck; Darmstadt, Germany).

### 4.3. Antibiotic Susceptibility Testing

Antibiotic susceptibility was tested by the disc-diffusion method on Mueller–Hinton agar (Kirby–Bauer method) to 13 antimicrobial agents. The following discs (Bio-Rad; Hercules, CA, USA) were used: ampicillin (AMP-10 μg), cefotaxime (CTA-30 μg), ceftiofur (CTF-30 μg) gentamicin (GEN-10 μg), neomycin (NEO-30 μg), streptomycin (STR-10 μg), tetracycline (TET-30 μg), doxycycline (DOX-30 μg), enrofloxacin (ENR-5 μg), nalidixic acid (NAL-30 μg), trimethoprim/sulfamethoxazole (TRS-23.75/1.25 μg), sulfonamide (SUL-200 μg), and chloramphenicol (CHL-30 μg). *E.* *coli* ATCC 25922 was used as a control strain. Colistin (COL) was not included due to its incompatibility with the disc-diffusion method, but it was tested by MIC determination using the broth microdilution reference method according to ISO 20776–1:2006. Data were interpreted using EUCAST epidemiological cut-off values (www.EUCAST.org).

### 4.4. Screening for Antibiotic Resistance Genes

After antimicrobial susceptibility testing, resistant strains were screened by PCR for putative determinants with primers and previously described experimental conditions. The following resistance genes were analyzed: *bla*_TEM_ [56], *bla*_OXA_ [57], *tetA* and *tetB* [58], *strA* and *strB* [59], *aadA1* [60], *aph2* [61], *sul1*, *sul2* and *sul3* [62], and *mcr-1* [63]. The Class 1 integrase gene (*int1*) was screened for in all isolates [64] and the presence of a variable region linked to the Class 1 integron was amplified by PCR and sequenced to determine the composition of its gene cassette [65].

## 5. Conclusions

*S.* Choleraesuis from IP and WB raised in close environments were found clonally related and transfer antimicrobial resistance determinants mainly by vertical transmission, whereas megaplasmids were detected linked to MDR, including colistin resistance in a single isolate carrying *mcr*-1. The role of *S.* Choleraesuis in the spread of antimicrobial resistance between wild and domestic swine should be carefully surveyed.

## Figures and Tables

**Table 1:** Antibiotic resistance characteristics of *S.* Choleraesuis isolates from Iberian pigs and wild boar in Spain.

PT ^1^	Isolate	Origin	Resistance Phenotype	Resistance Genotype	Plasmid Size (kb) ^2^
**1**	R145	WB	AMP–STR–TET–TRS–SUL	*bla*_TEM_ *–aadA1–sul1–sul3–tetA*	>105
	5662	IP	AMP–DOX–TRS–SUL–CHL	*bla*_TEM_ *–aadA1–sul3–Int1*	55
	6011	IP	AMP–STR–TRS–SUL	*bla*_TEM_	55
	6012	IP	AMP–STR	*bla*_TEM_	ND
	5661	IP	AMP–DOX	*bla*_TEM_	55
	5663	IP	AMP–STR–TRS–SUL	*bla*_TEM_	ND
	330	IP	AMP–GEN–NEO–STR–TET–DOX–TRS–SUL–COL	*strA–strB–sul1–mcr–1*	55 +> 244 ^3^
**2**	I 82	WB	AMP–NEO–TET–DOX	*bla*_TEM_	55
**3**	M1452	IP	AMP–NEO–STR–TET–DOX–TRS–SUL–CHL	*bla*_TEM_ *–tetA–Int1(aadA1)* ^4^	55
	I 144	WB	-	-	55
	I 160	WB	NEO	-	55
	I 163	WB	NEO	-	55
**4**	I 329	IP	AMP–STR–TET–DOX–TRS–SUL	*bla*_TEM_ *–strA–strB–sul1–Int1–(blaPSE1)* ^4^	55 + 244
**5**	R40	WB	STR–TET–DOX–SUL	*aadA1–strA–strB–sul1–tetA*	<33 + 55 + 310
	R160	WB	STR–TET–SUL	*strA–strB–tetA*	<33 + 55 + 240
	I 203	WB	STR–TRS–SUL	*strA–strB–tetA*	<33 + 55 + 310
**6**	5649	IP	AMP–TRS–SUL–CHL	*bla*_TEM_ *–aadA1–sul3–Int1*	105
	5650	IP	AMP–TRS–SUL–CHL	*bla*_TEM_ *–aadA1–sul3–Int1*	105
	5655	IP	AMP–TRS–SUL–CHL	*strA–sul3*	105
**7**	I 36	IP	AMP–NEO–STR–TET–DOX–SUL	*aadA1–strA–strB–sul2–tetB*	-

^1^ Pulsotype, as deduced from Figure 1. ^2^ DNA bands detected by PFGE-S1, with size (kb) deduced by proximity to corresponding bands in the *S. braenderup* standard; ^3^ hybridized to DIG-labeled *mcr-1*; ^4^ Genes identified in *int1*-linked gene cassettes. None detected. ND, not determined.

**Table 2:** Prevalence of antimicrobial resistance determinants among *S.* Choleraesuis isolates from Iberian pigs and wild boar in Spain.

Antimicrobials		IP		WB	
		N ^1^	Genes ^2^	N ^1^	Genes ^2^
**Sulfonamides**	Sulfadiazine	10	*sul1* (2), *sul2* (1), *sul3* (4)	4	*sul1* (2), *sul3* (1)
	Cotrimoxazol	9		2	
**β-lactams**	Ampicillin	12	*bla*_TEM_ (9), *bla*_PSE_ (1)	2	*bla*_TEM_ (2)
**Aminoglycosides**	Gentamycin	1	-	0	-
	Neomycin	3	-	3	-
	Streptomycin	7	*aadA* (5), *strA* (3), *strB* (3)	4	*aadA* (2), *strA* (3), *strB* (3)
**Tetracyclines**	Tetracycline	4	*tetA* (1), *tetB* (1)	4	*tetA* (4)
	Doxycycline	6		2	
**Phenicols**	Chloramphenicol	5		0	-
**Polymixins**	Colistin	1	*mcr-1* (1)	0	-

^1^ Number of isolates sharing resistance to indicated antimicrobial. ^2^ Number of resistance determinants between parenthesis. None detected.
